# Percentage of Body Fat Assessment Using Bioelectrical Impedance Analysis and Dual-Energy X-ray Absorptiometry in a Weight Loss Program for Obese or Overweight Chinese Adults

**DOI:** 10.1371/journal.pone.0058272

**Published:** 2013-04-01

**Authors:** Yi-Chun Li, Chia-Ing Li, Wen-Yuan Lin, Chiu-Shong Liu, Hua-Shui Hsu, Cheng-Chun Lee, Fei-Na Chen, Tsai-Chung Li, Cheng-Chieh Lin

**Affiliations:** 1 Graduate Institute of Biostatistics, College of Public Health, China Medical University, Taichung, Taiwan; 2 School of Medicine, College of Medicine, China Medical University, Taichung, Taiwan; 3 Department of Medical Research, China Medical University Hospital, Taichung, Taiwan; 4 Department of Family Medicine, China Medical University Hospital, Taichung, Taiwan; 5 Department of Neurology, China Medical University Hospital, Taichung, Taiwan; 6 Institute of Health Care Administration, College of Health Science, Asia University, Taichung, Taiwan; Universität Bochum, Germany

## Abstract

The current study aimed to compare the estimates of body fat percentage (%BF) by performing bioelectrical impedance analysis (BIA) and dual energy X-ray absorptiometry (DXA) in a sample of obese or overweight Chinese adults who participated in a weight-loss randomized control trial stratified by gender to determine whether or not BIA is a valid measurement tool. Among 189 adults [73 males, 116 females; age  = 41 to 74 years; mean body mass index (BMI)  = 27.3 kg/m^2^], assessments of %BF at the baseline and six months from the baseline were conducted by performing BIA and DXA. Bland-Altman analyses and multiple regression analyses were used to assess the relationships between %BF_BIA_ and %BF_DXA_. Compared with DXA, BIA underestimated %BF [in males: 4.6, –2.4 to 11.7 (mean biases, 95% limit of agreement) at the baseline, 1.4, –7.4 to 10.2 at the endpoint, and 3.2, –4.8 to 11.3 in changes; in females: 5.1, –2.4 to 12.7; 2.2, –6.1 to 10.4; and 3.0, –4.8 to 10.7, respectively]. For males and females, %BFDXA proved to be a significant predictor of the difference between DXA and BIA at the baseline, the endpoint, and in changes when BMI and age were considered (in males: p<0.01 and *R* ^2^  = 23.1%, 24.1%, 20.7%, respectively; for females: p<0.001 and *R* ^2^  = 40.4%, 48.8%, 25.4%, respectively). The current study suggests that BIA provides a relatively accurate prediction of %BF in individuals with normal weight, overweight, or obesity after the end of weight-loss program, but less accurate prediction of %BF in obese individuals at baseline or weight change during the weight-loss intervention program.

## Introduction

Obesity is a major global health problem, which affects people in developed and developing countries [1]–[4]. Approximately 250 million adults suffer from obesity [5], [6]. According to surveys, the age-adjusted prevalence of obesity for males is rapidly increasing in Taiwan from 10.5% to 15.9% from 1993 to 1996 and from 2000 to 2001 [7]. Obesity is also associated with numerous chronic health conditions or diseases, such as diabetes, hypertension, and cardiovascular diseases [8], [9].

The commonly used indicators for obesity are body mass index (BMI), waist circumference (WC), waist-height ratio (WHtR), and waist-to-hip ratio (WHR). Bioelectrical impedance analysis (BIA) produces a close estimate of fat mass in a wide range of body compositions [10]. BIA is a non-invasive measurement of body composition and particularly useful in large epidemiologic studies. BIA has also many advantages compared with other methods because it is inexpensive, simple, fast, safe, portable, and easy to perform, as well as requires minimum operator training [11], [12]. Instead of using the common indicators for obesity such as BMI, WC, WHtR, and WHR, BIA is used to determine the body fat percentage (%BF). Other obesity indicators do not measure %BF because of their inability to distinguish fat from muscle. For instance, a study has found that adiponectin, a substance secreted by the adipose tissue, can regulate energy homeostasis as well as glucose and lipid metabolism [13]. Adiponectin is also involved in inhibiting inflammatory responses through its inhibitory functions [14]. Another study has revealed that adiponectin is also associated with body fat distribution [15], even in normal-weight subjects [16]. Furthermore, %BF is more effective in detecting individuals with disturbed glucose tolerance, risk of cardiometabolic disease, early stage cardiovascular disease, and breast cancer survival rate [17]–[20]. Thus, %BF should be determined.

A previous study confirmed the validity of BIA in estimating %BF compared with dual-energy X-ray absorptiometry (DXA) [12]. DXA is a valid method to aid studies on obesity [21]. However, radiation emitted during DXA measure has been reported in previous studies [22]–[24]. The effective radiation dose of DXA is among the lowest from the commonly used medical X-ray examinations and lower than a standard chest X-ray [22]. Therefore, DXA is appropriate for clinical patient treatment; however, unlike BIA, DXA limits epidemiological studies and wide application. BIA is a good predictor of DXA-derived fat-free mass (*r* = 0.85 to 0.88) [12]. BIA measurements are determined by the resistance of the body to electrical current flow between points of contact on the body and correlates well with total body water measurements [25]. BIA exhibits greater resistance to electrical current flow in fat tissues than fat-free tissues because of their differences in water content.

Several studies have been conducted on the validation of BIA in the estimation of %BF compared with DXA [12], [26]–[36]. However, only two %BF studies, in which BIA was used, has been conducted among Asian adults. One study [29] involves a cross-sectional investigation stratified by gender and the other [36] uses a longitudinal study on Korean women. Most of the studies have been conducted on Caucasians [26], [28], [30]–[32], [34] and African-Americans [27], [33]. Several studies have considered only women [27], [30], [32], [33], [36] or adolescents [26], [27]. Longitudinal studies [30], [34]–[37] have also been performed, in which two studies have used weight-loss programs without randomization [34], [36]. The sample sizes used in most of these studies are small, which range from 53 to 136 subjects [26]–[29], [31]–[33]. Only two studies have considered a large sample size (*n*>500) [12], [34]. The sample sizes of previous longitudinal studies [30], [34]–[37] ranged from 34 to 105 subjects. The validation of the hand-to-foot BIA with simple frequency is also limited [29], [32].

Addressing questions on whether or not BIA is a valid method for monitoring the changes in %BF to evaluate the effects of weight-loss programs is highly important because of the increasing need for an administrable and accurate instrument in weight-loss programs.

The current study aimed to compare estimates of %BF by performing BIA and DXA in a sample of obese or overweight Chinese adults who participated in a weight-loss intervention trial in Taiwan. This study also determined whether or not BIA is a valid measurement tool for weight-loss programs.

## Materials and Methods

### Subjects

The study population consisted of obese or overweight individuals who are in good health and reside in the Taichung city, which is located in west-central Taiwan. Data were collected from participants of a randomized intervention study, in which the effects of three weight-loss programs were evaluated. The duration of the intervention programs was six months. Assessments were made at the baseline and six months from the baseline. The subjects were recruited during community screening activities, advertisements, and Internet propaganda. Before randomization, 240 obese or overweight adults (age >40 years) at the baseline were recruited according to either one of the two criteria of obesity: (1) BMI recommended by the Department of Health, Executive Yuan, R.O.C [38] and (2) WC recommended by an Asian modification for the (central) obesity by National Cholesterol Education program Adult Treatment Panel-III (NCEP ATP-III) definition of metabolic syndrome (MetS) [39]. The BMI cut-off points for obese and overweight were 27 and 24 kg/m^2^, respectively. The WC cut-off points for (central) obesity were 90 and 80 cm in males and females, respectively. Under this condition, several subjects with (central) obesity determined by WC at the baseline may have a normal BMI. Complete data included 189 subjects (73 males and 116 females); 51 subjects were removed because of missing or incomplete data at the end of the study. The study was approved by the Human Research Committee of the China Medical University Hospital. Written informed consent was obtained from each participant.

### Anthropometric measurements

Weight and height were measured using an auto-anthropometer (Super-View, HW-666, Taipei, Taiwan) and estimated to the nearest 0.1 kg and 0.1 cm, respectively (the subjects were shoeless and wore light clothing). BMI was calculated as weight (kg) divided by the square of height (m). %BF was determined by performing BIA and DXA.

### BIA

A body composition analyzer (Tanita BC-418, Tanita Corp., Tokyo, Japan) was used to estimate %BF. Eight polar electrodes were applied on both feet and both hands. The electric current was then supplied from the electrodes on the tips of the toes and fingers and the voltage was measured on the heel of both feet and the thenar side of both hands. The analyzer provided a complete %BF analysis, including %BF, in less than 30 s. The analyzer can be used for subjects with weights of up to 200 kg and measure the body composition by using a constant current source at a frequency current of 50 kHz (500 µÅ). Before the assessment, the participants were subjected to a 12 h fasting period and instructed to avoid heavy physical activity, alcohol ingestion, and diuretic intake. After the assessors manually documented the weight of clothes, body type, age, and height into the system, the subjects in light clothing removed their shoes, wiped their feet, and stood on the weighing platform without bending their knees. Measurement began when the subjects placed their hands on the grips. Thereafter, the process was completed. The BIA variable used in the present study was %BF_BIA_.

### DXA

Fat mass was measured using DXA (GE-LUNAR DPX PRO, Lunar Corp., Madison, WI, USA). Data were analyzed using the enCORE2004 software, version 8.60.006 at coefficient of variation <1%. The subjects were subjected to DXA in their underwear, without any metal items. The operator performed a whole-body scan on each subject as he/she lies in a supine position. Whole-body composition analysis provided data on different regions of interest, including the trunk, arms, and legs. Equipment was calibrated each day by using a standardized phantom. The DXA variable used in the present study was %BF_DXA_.

### Statistical analysis

Descriptive data at the baseline, the endpoint, and in changes stratified by gender were presented as mean (SD). The methods used to assess the relationships between %BF_BIA_ and %BF_DXA_ stratified by gender at different stages were Pearson's correlation coefficients (*r*), Bland-Altman analyses, and multiple regression analyses. *r* among BMI, %BF_BIA_, and %BF_DXA_ were also calculated. The level of correlation was considered low or weak when *r*≤0.35, moderate when *r* = 0.36 to 0.67, and strong when *r* = 0.68 to 1.0 [40]. The mean bias and 95% limits of agreement between %BF_BIA_ and %BF_DXA_ were estimated in the Bland-Altman plots and reported by table, with the %BF_DXA_ as the gold standard and %BF_BIA_ as the comparison. Stepwise multiple regression analyses were used to identify the significant predictors of differences between %BF_BIA_ and %BF_DXA_. A significant explanatory variable for this difference indicates that the measurement error systematically varied according to the values of this explanatory variable. Thus, the measurement errors were associated with this explanatory variable. Under this condition, the explanatory variable is a measurement bias. However, an explanatory variable is not a significant predictor, indicating that the measurement error is random according to this explanatory variable. The higher *R* ^2^ and the greater magnitude of the difference between %BF_BIA_ and %BF_DXA_ can be explained by the explanatory variables. The first model includes %BF_DXA_, whereas the second model considers %BF_DXA_, BMI, and age. All of the analyses were performed using SAS version 9.2 (SAS Institute, Cary, North Carolina). All two-sided p-values <0.05 were considered statistically significant.

## Results

### Study subject characteristics

Table 1 presents the baseline characteristics and the subjects' subsequent changes by gender. Their ages ranged from 41 to 74 years. Based on BMI, male subjects who were obese, overweight, and had normal weight at the baseline were 46.6%, 52.1%, and 1.4%, respectively. In females, the corresponding percentages were 38.8%, 50.9%, and 10.3%, respectively. Females had a higher percentage of total body fat in BIA and DXA. During the intervention period, decreases in weight, BMI, and %BF_DXA_ were observed, but a slight increase in %BF was found in BIA at the endpoint. After the intervention program, 13.76% of the subjects had normal weight, in which eight were male and eight were female. For both males and females, BIA underestimated %BF with the greatest difference at the baseline and the least difference at the endpoint.

**Table 1:** Subject characteristics.

Gender	Variable	Baseline	Endpoint	Change
**Male**	AGE (year)	52.8(7.0)		
(n = 73)	HEIGHT (cm)	68.0(6.0)		
	WEIGHT (kg)	78.3(10.3)	76.5(10.6)	1.9(2.8)
	BMI (kg/m^2^)	27.7(2.6)	27.0(2.7)	0.68(1.1)
	%BF_BIA_ (%)	25.3(4.4)	26.8(5.8)	−1.5(4.7)
	%BF_DXA_ (%)	29.9(3.4)	28.1(4.5)	1.7(2.6)
	Δ%BF (%)	4.6(3.6)	1.4(4.5)	3.2(4.1)
	MEAN %BF (%)	27.6(3.5)	27.4(4.7)	0.12(3.2)
	ICC (%BF)	0.58	0.63	0.41
				
**Female**	AGE (year)	52.4(6.9)		
(n = 116)	HEIGHT (cm)	155.5(5.5)		
	WEIGHT (kg)	65.4(8.2)	62.7(8.1)	2.7(3.4)
	BMI (kg/m^2^)	27.0(3.0)	25.9(3.1)	1.1(1.4)
	%BF_BIA_ (%)	36.6(4.4)	37.3(5.5)	−0.68(4.3)
	%BF_DXA_ (%)	41.7(4.6)	39.4(5.5)	2.3(3.2)
	Δ%BF (%)	5.1(3.8)	2.2(4.2)	3.0(4.0)
	MEAN %BF (%)	39.1(4.1)	38.3(5.1)	0.80(3.2)
	ICC (%BF)	0.64	0.71	0.45

Values are mean(SD); BMI, body mass index; %BF, percentage of body fat; DXA.

dual energy x-ray absorptiometry; BIA, bioelectrical impedance analysis equipment.

Δ%BF, mean difference between methods (%BFDXA-%BFBIA); MEAN %BF mean of methods (%BFDXA, %BFBIA). ICC, intraclass correlation coefficient.

### Agreement between methods

**Figure 1:**
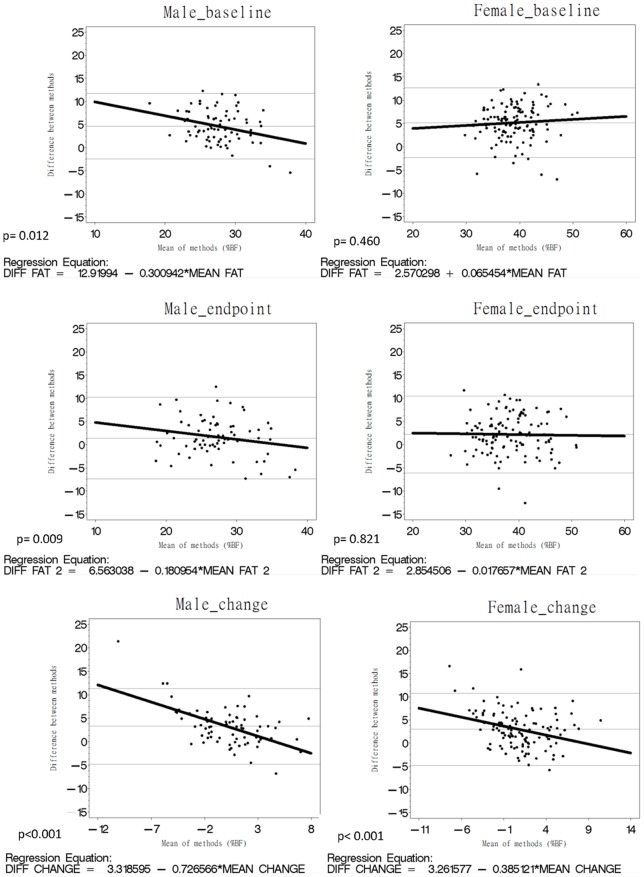
Bland-Altman plots with mean bias (central line) and 95% limits of agreement for comparing %BF_BIA_ and %BF_DXA_ at the baseline, endpoint, and in changes. The central line represents the mean bias between %BF_BIA_ and %BF_DXA_; the outer lines represent 95% limits of agreement.

For males or females, %BF and BMI were significantly correlated (Table 2). Three strong *r* were observed in females, particularly between %BF_BIA_ and BMI, between %BF_BIA_ and %BF_DXA_ at the endpoint, as well as between %BF_DXA_ and BMI in changes. Agreement between these methods is shown in Table 3 and Figure 1 by using the Bland-Altman plots with mean bias and 95% limits of agreement. For both males and females, the mean differences at the endpoint were the smallest compared with the baseline and in changes (Table 3). The slopes in Figure 1 show that the correlation between the differences in DXA and BIA, as well as the mean %BF measured using both methods was stronger in males than that in females at the baseline, the endpoints, and in change. The lines at the endpoint were more parallel to the *x*-axis than those at the baseline and in changes in males and females. At the endpoint, the magnitude of the underestimation of %BF based on BIA decreased compared with that at the baseline. Table 3 shows that the mean difference between %BF_DXA_ and %BF_BIA_ was larger in females than that in males at the baseline and the endpoint, but slightly smaller in males than that in females at changes. Table 4 reveals that the magnitude of difference between %BF_DXA_ and %BF_BIA_ based on DXA, age, and BMI was greater in females than that in males (*R* ^2^_female_  = 0.23 vs. *R* ^2^_male_  = 0.05 at the baseline; *R* ^2^_female_  = 0.13 vs. *R* ^2^_male_  = 0.03 at the endpoint; and *R* ^2^_female_  = 0.09 vs. *R* ^2^_male_  = 0.01 in change).

**Table 2:** Pearson correlation matrix for body fat percentage. and anthropometric variables.

	Male (n = 73)		Female (n = 116)	
	%BF_BIA_	%BF_DXA_	%BFBIA	%BF_DXA_
Baseline				
BMI	0.61	0.54	0.65	0.59
%BF_BIA_		0.60		0.64
Endpoint				
BMI	0.65	0.65	0.81	0.66
%BF_BIA_		0.65		0.71
Change				
BMI	0.58	0.65	0.58	0.79
%BF_BIA_		0.48		0.47

All are significant at the p<0.001 level.

**Table 3:** Mean differences and 95% limits of agreement between %BF_BIA_ and %BF_DXA_ using Bland-Altman analysis.

	Male (n = 73)		Female (n = 116)	
	Mean difference	95% limits of agreement	Mean difference	95% limits of agreement
Baseline	4.6	−2.4 to 11.7	5.1	−2.4 to 12.7
Endpoint	1.4	−7.4 to 10.2	2.2	−6.1 to 10.4
Change	3.2	−4.8 to 11.3	3.0	−4.8 to 10.7

**Table 4:** Regression analysis of the difference between DXA and BIA.

Δ%BF	Explanatory variables	Parameter Estimate	p value	R^2^
Male				
Baseline	%BF_DXA_	0.23	0.06	4.9%
	%BF_DXA_	0.53	<.001	23.1%
	BMI	0.72	<.001	
	AGE	−0.04	0.51	

Endpoint	%BF _DXA_	0.18	0.13	3.3%
	%BF _DXA_	0.59	<.001	24.1%
	BMI	−0.96	<.001	
	AGE	−0.16	0.02	

Change	%BF _DXA_	0.15	0.41	0.98%
	%BF _DXA_	0.72	0.002	20.7%
	BMI	−2.0	<.001	
	AGE	0.11	0.10	

Female				
Baseline	%BF _DXA_	0.40	<.001	22.8%
	%BF _DXA_	0.65	<.001	40.4%
	BMI	−0.62	<.001	
	AGE	0.08	0.05	

Endpoint	%BF _DXA_	0.28	<.001	13.5%
	%BF _DXA_	0.69	<.001	48.8%
	BMI	−1.1	<.001	
	AGE	−0.01	0.83	

Change	%BF _DXA_	0.38	<.001	9.4%
	%BF _DXA_	0.99	<.001	25.4%
	BMI	−1.8	<.001	
	AGE	0.08	0.11	

Table 4 also shows the statistical analysis for the regression of the differences between DXA and BIA on explanatory variables at the baseline, the endpoint, and in changes stratified by gender. In males, %BF_DXA_ alone was not a significant predictor for Δ%BF at the baseline, the endpoint, and in changes. The corresponding percentages of variation for the differences between DXA and BIA explained by %BF_DXA_ were 4.9%, 3.3%, and 0.98% at the baseline, the endpoint, and in changes, respectively. BMI and age resulted in significant %BF_DXA_ in all of the models. In addition to %BF_DXA_, BMI was a significant predictor in all of the models. Age was only significant in the model at the endpoint. The corresponding percentages of variation for the difference between DXA and BIA based on %BF_DXA_, BMI, and age were 23.1%, 24.1%, and 20.7%, respectively. In females, %BF_DXA_ was a significant predictor for Δ%BF at the baseline, the endpoint, and in changes. The corresponding percentages of variation for the difference between DXA and BIA based on %BF_DXA_ at the baseline, the endpoint, and in changes were 22.8%, 13.5%, and 9.4%, respectively. %BF_DXA_ remained significant in all of the models when BMI and age were considered. In addition to %BF_DXA_, BMI was significant in all of the models. The corresponding percentages of variation for the differences between DXA and BIA based on %BF_DXA_, BMI, and age were 40.4%, 48.8%, and 25.4%, respectively.

## Discussion

The current study showed that BIA is a useful measuring tool for the assessment of %BF at the endpoint in individuals with normal weight, overweight, or obesity, but provides less accurate predictions of %BF in changes during the weight-loss intervention programs. This result was demonstrated by the strong correlation between %BF parameters determined by BIA and DXA. Our Bland-Altman agreement analysis showed that BIA underestimated %BF. This bias increased as %BF_DXA_ increased. The variation in percentage of this bias, which is associated with %BF_DXA_, was significantly higher in females than that in males. Therefore, caution should be used when interpreting the changes in %BF between the baseline and the endpoint when BIA is used in clinical practice, particularly in obese or overweight Chinese females.

Several studies have compared BIA with DXA as the reference method. However, results are mixed. In general, when assessing %BF in cross-sectional studies, some studies have reported that BIA overestimates %BF or fat mass weight [28], [34], [36], whereas others have revealed that BIA underestimates %BF or fat mass weight [26], [27], [30], [33], which is also found in the present study. Similarly, among these longitudinal studies, some have underestimated %BF at the baseline, at the endpoint, and in change, whereas some studies have overestimated %BF at different time points. However, results in previous studies are difficult to compare with those in the present study because various devices were used, such as foot-to-foot BIA [26], [34], devices with adhesive tape [26], [27], [30], [33], [35], [36], or multi-frequency devices [28]. Our results are in agreement with those in two cross-sectional studies, in which a BIA-type device, namely, the hand-to-foot BIA with simple frequency, was used. In particular, similar estimates of %BF have been reported [29], [32]. In one of these two studies, 72 obese Japanese adults (age  = 51.2±9.4 years; BMI  = 28.0±2.2 kg/m^2^) were considered, in which body fat at the extremities, trunk, and total body was determined using DXA and single-frequency BIA equipment (BC-118, Tanita Corp., Tokyo, Japan) [29]. The accuracy of the estimated %BF value in the trunk was lower than that of the total body. SF-BIA reveals an underestimated %BF in the total body in males. In another study, Neovius et al. also examined the accuracy of SF-BIA (BC-418, Tanita Corp., Tokyo, Japan) in 136 obese Swedish women (age  = 48.1±7.7 years; BMI: 30.4±2.9 kg/m^2^) [32]. The BIA equipment used has revealed an underestimated %BF, which is consistent with our findings. Two possible reasons can explain this underestimated result. First, 50 kHz of current can pass through extracellular and intracellular spaces, and thus individual hydration states can be used as a factor of error. Second, the body position, particularly during standing, can change the fluid distribution, which can influence the measurement of resistance.

Our regression analysis indicated that a higher %BF_DXA_ increased the biases between DXA and BIA at the baseline, the endpoint, and in changes in males and females, and a higher BMI decreased the biases, except BMI at the baseline in males. These results are consistent with those of Neovius et al. [32], in which the variation in percentages for the differences between DXA and BIA based on %BF_DXA_ was similar to that in our study at the baseline in females. However, Neovius only evaluated the performance of BIA compared with DXA in centrally obese women by using a cross-sectional study [32]. Our findings further revealed the changes in obese individuals during the intervention programs. The results in males and females with normal weight, overweight, or obesity were also reported. Our findings indicated that biases were significantly greater in females compared with those in males, which is attributed to the regional differences in the fat distribution in the body.

Few studies have investigated this issue in Asia. In addition to the findings in the previous Japanese report [29], a Korean longitudinal study compared the body composition assessments in terms of BIA and DXA before and after a six-week herbal diet intervention program in 50 pre-menopausal women (age  = 30.6±6.2 years; BMI  = 31.7±3.8 kg/m^2^) [36]. In these two studies, *r* between %BF_BIA_ and %BF_DXA_ is significant [29], [36], which is consistent with our findings. However, *r* is slightly higher in Japanese (*r*>0.70, p<0.01) and Koreans before the six-week intervention (*r* = 0.79, *p*<0.05) compared with that in our findings (*r* = 0.64 at the baseline; *r* = 0.71 at the endpoint, p<0.001) in the female group. Differences between BIA and DXA measurements were also observed in these two studies [29], [36]. In our study, BIA underestimated %BF of the total body in both genders, but was only observed in males in the previous Japanese study [29]. By contrast, BIA overestimated the total body fat by 2.54 kg and changes in %BF compared with DXA (p<0.001) in the Korean study [36]. Thus, we need a larger sample size to obtain more accurate information in future studies for a better understanding of the differences between BIA and DXA.

The present study has both strengths and weaknesses. For the strengths, our sample size was larger compared with previous longitudinal studies or randomized control trials that having size of samples from 34 to 136. Due to the larger sample size, an evaluation stratified by genders was available in the present study. Our study also evaluated the accuracy of BIA among obese or overweight subjects, and the endpoint of the study allowed us to estimate the accuracy of BIA among subjects with normal weight, overweight, or obesity after participating in the weight-loss programs. The current study also described the subjects at different periods; thus, we can determine the accuracy of BIA in weight changes during the intervention programs.

The current study also has several limitations. The use of pencil-beam DXA as the gold standard was the first limitation because the device is an older generation of DXA instrument. A previous study compared the %BF measurements between pencil-beam and fan-beam DXA [41]. Results showed that the mean differences were –0.7% for %fat and 6.4% for fat in kg, indicating that the two DXA technologies can be considered equivalent. We also used the same instrument in %BF during the entire intervention period, which prevented extra variations and minimized bias to evaluate the accuracy of BIA measurements. Second, we only studied Chinese adults. Ethnic- and age-related differences in the accuracy of BIA measurements likely exist. Studies of other ethnic groups, children, or adolescents may yield different results. Third, we did not include a severely obese group; we only tested the accuracy in the overall excess weight. Therefore, our results were limited to a severely obese cohort.

In conclusion, BIA is useful and relatively cheap equipment in the assessment of obesity in both genders. During the weight-loss program period, we found that BIA provides a valid estimate at the endpoint, particularly in individuals with normal weight, overweight, or obesity, but provides less accurate predictions of %BF at the baseline in obese individuals or in weight changes during the intervention programs. This study is the first to validate BIA in a randomized control trial of weight loss in Chinese subjects. Our findings indicate that BIA underestimated %BF. The difference between DXA and BIA is likely to increase as an individual's %BF_DXA_ increases. Thus, this study should be replicated using other ethnic and age groups with a severely obese cohort.

## References

[1] Kannel WB, d'Agostino R, Cobb JL (1996) Effect of weight on cardiovascular disease. Am J Clin Nutr 63: 419S–422S. 8615332 10.1093/ajcn/87.6.1602

[2] Carroll K (1998) Obesity as a risk factor for certain types of cancer. Lipids 33: 1055–1059. 9870899 10.1007/s11745-998-0305-8

[3] Alberti K, Zimmet P (1998) Definition, diagnosis and classification of diabetes mellitus and its complications. Part 1: diagnosis and classification of diabetes mellitus. Provisional report of a WHO consultation. Diabet Med 15: 539–553. 9686693 10.1002/(SICI)1096-9136(199807)15:7<539::AID-DIA668>3.0.CO;2-S

[4] Consultation W (2000) Obesity: preventing and managing the global epidemic. World Health Organ Tech Rep Ser 894: i-xii, 1–253. 11234459

[5] Kuczmarski RJ, Flegal KM, Campbell SM, Johnson CL (1994) Increasing prevalence of overweight among US adults. JAMA 272: 205–211. 8022039 10.1001/jama.272.3.205

[6] Seidell JC, Verschuren W, van Leer EM, Kromhout D (1996) Overweight, underweight, and mortality: a prospective study of 48287 men and women. Arch Intern Med 156: 958–963. 8624176 10.1001/archinte.156.9.958

[7] Chu NF (2005) Prevalence of obesity in Taiwan. Obes Rev 6: 271–274. 16246212 10.1111/j.1467-789X.2005.00175.x

[8] Must A, Spadano J, Coakley EH, Field AE, Colditz G, et al (1999) The disease burden associated with overweight and obesity. JAMA 282: 1523–1529. 10546691 10.1001/jama.282.16.1523

[9] Kopelman PG (2000) Obesity as a medical problem. Nature 404: 635–643. 10766250 10.1038/35007508

[10] Deurenberg P, Van der Kooy K, Leenen R, Weststrate J, Seidell J (1991) Sex and age specific prediction formulas for estimating body composition from bioelectrical impedance: a cross-validation study. Int J Obes 15: 17–25. 2010255

[11] Houtkooper LB, Lohman TG, Going SB, Howell WH (1996) Why bioelectrical impedance analysis should be used for estimating adiposity. Am J Clin Nutr 64: 436S–448S. 8780360 10.1093/ajcn/64.3.436S

[12] Roubenoff R (1996) Applications of bioelectrical impedance analysis for body composition to epidemiologic studies. Am J Clin Nutr 64: 459S–462S. 8780363 10.1093/ajcn/64.3.459S

[13] Yamauchi T, Kamon J, Minokoshi Y, Ito Y, Waki H, et al (2002) Adiponectin stimulates glucose utilization and fatty-acid oxidation by activating AMP-activated protein kinase. Nat Med 8: 1288–1295. 12368907 10.1038/nm788

[14] Yokota T, Oritani K, Takahashi I, Ishikawa J, Matsuyama A, et al (2000) Adiponectin, a new member of the family of soluble defense collagens, negatively regulates the growth of myelomonocytic progenitors and the functions of macrophages. Blood 96: 1723–1732. 10961870

[15] Staiger H, Tschritter O, Machann J, Thamer C, Fritsche A, et al (2003) Relationship of serum adiponectin and leptin concentrations with body fat distribution in humans. Obes Res 11: 368–372. 12634431 10.1038/oby.2003.48

[16] Festen DA, van Toorenenbergen A, Duivenvoorden HJ, Hokken-Koelega AC (2007) Adiponectin levels in prepubertal children with Prader-Willi syndrome before and during growth hormone therapy. J Clin Endocrinol Metab 92: 1549–1554. 17264186 10.1210/jc.2006-2241

[17] Gómez-Ambrosi J, Silva C, Galofré JC, Escalada J, Santos S, et al (2011) Body adiposity and type 2 diabetes: increased risk with a high body fat percentage even having a normal BMI. Obesity 19: 1439–1444. 21394093 10.1038/oby.2011.36

[18] Shea JL, King MT, Yi Y, Gulliver W, Sun G (2012) Body fat percentage is associated with cardiometabolic dysregulation in BMI-defined normal weight subjects. Nutr Metab Cardiovasc Dis 22: 741–747. 21215604 10.1016/j.numecd.2010.11.009

[19] Yamashita K, Kondo T, Osugi S, Shimokata K, Maeda K, et al (2012) The Significance of Measuring Body Fat Percentage Determined by Bioelectrical Impedance Analysis for Detecting Subjects with Cardiovascular Disease Risk Factors. Circ J 76: 2435–2442. 22784998 10.1253/circj.cj-12-0337

[20] Dee A, McKean-Cowdin R, Neuhouser ML, Ulrich C, Baumgartner RN, et al (2012) DEXA measures of body fat percentage and acute phase proteins among breast cancer survivors: a Cross-Sectional Analysis. BMC Cancer 12: 343. 22873489 10.1186/1471-2407-12-343 PMC3500231

[21] Evans EM, Misic MM, Mallard DM (2010) A technique to assess body composition and sarcopenia using DXA: application for an obese population. Eur J Clin Nutr 64: 218–220. 19904294 10.1038/ejcn.2009.128

[22] De Schepper J, Roggen I, Van Biervliet S, Robberecht E, Gies I, et al (2012) Comparative bone status assessment by dual energy X-ray absorptiometry, peripheral quantitative computed tomography and quantitative ultrasound in adolescents and young adults with cystic fibrosis. J Cyst Fibros 11: 119–124. 22119452 10.1016/j.jcf.2011.10.004

[23] Liu CR, Niu HJ, Pu F, Wang L, Sun LW, et al (2012) The effect of physical loading on calcaneus quantitative ultrasound measurement: a cross-section study. BMC musculoskel dis 13: 70. 10.1186/1471-2474-13-70 PMC3436732 22584084

[24] Xu B, Yu W, Yao M, Yao X, Li Q, et al (2009) A 3D surface imaging system for assessing human obesity. SPIE 7443: 74431U–12. 10.1117/1.3250191 PMC2788969 19966948

[25] Erceg DN, Dieli-Conwright CM, Rossuello AE, Jensky NE, Sun S, et al (2010) The Stayhealthy bioelectrical impedance analyzer predicts body fat in children and adults. Nutr Res 30: 297–304. 20579521 10.1016/j.nutres.2010.04.009

[26] Lazzer S, Boirie Y, Meyer M, Vermorel M (2003) Evaluation of two foot-to-foot bioelectrical impedance analysers to assess body composition in overweight and obese adolescents. Br J Nutr 90: 987–992. 14667192 10.1079/bjn2003983

[27] Newton R, Alfonso A, White M, York-Crowe E, Walden H, et al (2005) Percent body fat measured by BIA and DEXA in obese, African-American adolescent girls. Int J Obes 29: 594–602. 10.1038/sj.ijo.0802968 15889118

[28] Shafer KJ, Siders WA, Johnson LAK, Lukaski HC (2009) Validity of segmental multiple-frequency bioelectrical impedance analysis to estimate body composition of adults across a range of body mass indexes. Nutrition 25: 25–32. 18723322 10.1016/j.nut.2008.07.004

[29] Sato S, Demura S, Kitabayashi T, Noguchi T (2007) Segmental body composition assessment for obese Japanese adults by single-frequency bioelectrical impedance analysis with 8-point contact electrodes. J Physiol Anthropol 26: 533–540. 18092509 10.2114/jpa2.26.533

[30] Verdich C, Barbe P, Petersen M, Grau K, Ward L, et al (2011) Changes in body composition during weight loss in obese subjects in the NUGENOB study: Comparison of bioelectrical impedance vs. dual-energy X-ray absorptiometry. Diabetes Metab 37: 222–229. 21236715 10.1016/j.diabet.2010.10.007

[31] Bosy-Westphal A, Later W, Hitze B, Sato T, Kossel E, et al (2008) Accuracy of bioelectrical impedance consumer devices for measurement of body composition in comparison to whole body magnetic resonance imaging and dual X-ray absorptiometry. Obes Facts 1: 319–324. 20054195 10.1159/000176061 PMC6452160

[32] Neovius M, Hemmingsson E, Freyschuss B, Uddén J (2006) Bioelectrical Impedance Underestimates Total and Truncal Fatness in Abdominally Obese Women. Obesity 14: 1731–1738. 17062802 10.1038/oby.2006.199

[33] Newton Jr R, Alfonso A, York-Crowe E, Walden H, White M, et al (2006) Comparison of body composition methods in obese African-American women. Obesity (Silver Spring) 14: 415–422. 16648612 10.1038/oby.2006.55

[34] Lloret Linares C, Ciangura C, Bouillot JL, Coupaye M, Declèves X, et al (2011) Validity of Leg-to-Leg Bioelectrical Impedance Analysis to Estimate Body Fat in Obesity. Obes Surg 21: 1–7. 20936394 10.1007/s11695-010-0296-7

[35] Aslam M, Eckhauser AW, Dorminy CA, Dossett CM, Choi L, et al (2009) Assessing body fat changes during moderate weight loss with anthropometry and bioelectrical impedance. Obes Res Clin Pract 3: 209–219. 20161645 10.1016/j.orcp.2009.03.005 PMC2818292

[36] Kim H, Gallagher D, Song M (2005) Comparison of body composition methods during weight loss in obese women using herbal formula. Am J Chin Med 33: 851–858. 16355441 10.1142/S0192415X05003454

[37] Frisard M, Greenway F, Delany J (2005) Comparison of methods to assess body composition changes during a period of weight loss. Obes Res 13: 845–854. 15919837 10.1038/oby.2005.97

[38] Department of Health, Executive Yuan, R.O.C. (2002) The definition of obesity and intervention principles toward compatriots. Aug. 09. Available at: http://www.doh.gov.tw/CHT2006/DM/DM2_p01.aspx?class_no=25&now_fod_list_no=3942&level_no=2&doc_no=32 Accessed 09 November 2012.

[39] Crepaldi G, Maggi S (2006) The metabolic syndrome: a historical context. Diabetes voice 51: 8–10.

[40] Taylor R (1990) Interpretation of the correlation coefficient: a basic review. JDMS 6: 35–39.

[41] Ellis K, Shypailo R (1998) Bone mineral and body composition measurements: cross-calibration of pencil-beam and fan-beam dual-energy X-ray absorptiometers. J Bone Miner Res 13: 1613–1618. 9783550 10.1359/jbmr.1998.13.10.1613

